# Securing Resection Margin Using Indocyanine Green Diffusion Range on Gastric Wall during NIR Fluorescence-Guided Surgery in Early Gastric Cancer Patients

**DOI:** 10.3390/cancers14215223

**Published:** 2022-10-25

**Authors:** Minah Cho, Ki-Yoon Kim, Sung Hyun Park, Yoo Min Kim, Hyoung-Il Kim, Woo Jin Hyung

**Affiliations:** 1 Department of Surgery, Yonsei University College of Medicine, Seoul 03722, Korea; 2 Gastric Cancer Center, Yonsei Cancer Center, Yonsei University Health System, Seoul 03722, Korea

**Keywords:** early gastric cancer, gastrectomy, tumor localization, resection margin, near-infrared imaging, indocyanine green, fluorescent

## Abstract

**Simple Summary:**

A retrospective analysis of 503 patients with early gastric cancer located in the body of the stomach who underwent fluorescence lymphography-guided gastrectomy revealed that the indocyanine green (ICG) diffusion area along the gastric wall secured a resection margin of >28 mm. The ICG diffusion range can be used as a simple and easy method for determining the resection margin during gastrectomy using near-infrared (NIR) imaging.

**Abstract:**

Near-infrared (NIR) fluorescence lymphography-guided minimally invasive gastrectomy using indocyanine green (ICG) is employed to visualize draining lymphatic vessels and lymph nodes. Endoscopically injected ICG spreads along the gastric wall and emits fluorescence from the serosal surface of the stomach. We aimed to assess the efficacy of ICG diffusion in securing the resection margin. We retrospectively analyzed 503 patients with early gastric cancer located in the body of the stomach who underwent fluorescence lymphography-guided gastrectomy from 2018 to 2021. One day before surgery, ICG was endoscopically injected into four points of the submucosal layer peritumorally. We measured the extent of resection and the resection line based on the ICG diffusion area from the specimen using NIR imaging. The mean area of the ICG diffusion was 82.7 × 75.3 and 86.7 × 80.2 mm^2^ on the mucosal and serosal sides, respectively. After subtotal gastrectomy, the length of the proximal resection margin was 38.1 ± 20.1, 33.4 ± 22.2, and 28.7 ± 17.2 mm in gastroduodenostomy, loop gastrojejunostomy, and Roux-en-Y gastrojejunostomy, respectively. The ICG diffusion area along the gastric wall secured a resection margin of >28 mm. The ICG diffusion range can be used as a simple and easy method for determining the resection margin during gastrectomy using NIR imaging.

## 1. Introduction

Gastrectomy with adequate margins and systemic lymphadenectomy is the optimal treatment for patients with early gastric cancer (EGC) who require gastrectomy and for whom endoscopic submucosal resection is not indicated. Based on recent results from randomized clinical trials [1,2,3,4,5,6], minimally invasive surgery is acceptable as a standard option for EGC [7,8]. Hence, the proportion of gastric cancers treated using minimally invasive surgery has markedly increased over the past decades [9,10]. Most minimally invasive gastrectomies are performed with intracorporeal reconstruction, as intracorporeal anastomosis has benefits over extracorporeal anastomosis. However, EGC cannot be recognized from the serosal surface, and it is impossible to palpate EGC lesions, even during open surgery [7,8,11]. Thus, the exact tumor localization of EGC is essential to determine the gastric resection line, especially when performing distal gastrectomy with intracorporeal reconstruction. Various methods have been devised to locate early gastric cancer lesions during surgery; however, each method has various disadvantages [12,13]. Considering that most of these detection methods must be performed during surgery, which may increase operating time and require the use of specific equipment and additional personnel in the operating room, there is a significant amount of room for improvement.

Recently, near-infrared (NIR) fluorescence-guided surgery has been widely employed for minimally invasive gastrectomy to visualize lymphatic drainage from the primary tumor by endoscopic submucosal indocyanine green (ICG) injection around the tumor in four quadrants [14,15,16,17,18,19,20]. In particular, since the enlargement of metastatic lymph nodes is rare in early gastric cancer, fluorescence-guided lymphadenectomy is expected to reduce the risk of missing small, potentially metastatic lymph nodes by visualizing lymphatic structures draining from the tumor. As endoscopically injected ICG spreads along the gastric wall, the diffusion area emits NIR fluorescence from the serosal surface of the stomach, enabling visualization of the injection site during surgery. This phenomenon enables real-time identification of tumor location through the ICG diffusion area on the gastric wall using NIR fluorescence imaging without any additional procedures. Hence, we aimed to evaluate the efficacy of the ICG diffusion area for tumor localization by assessing the length of the ICG diffusion range and the distance of the proximal and distal resection margins during NIR fluorescence-guided gastrectomy in patients with clinical EGC.

## 2. Materials and Methods

### 2.1. Patients

This study was a retrospective analysis of prospectively collected gastric cancer data from January 2018 to December 2021. Patients diagnosed with an EGC located in the gastric body, patients who underwent submucosal ICG injection one day before surgery and NIR fluorescence-guided gastrectomy, and patients whose diffusion range was measured on the surgical specimen after surgery were included in the study. Patients who underwent additional surgery after endoscopic submucosal dissection and those with multiple synchronous gastric cancers were excluded in order to accurately evaluate the diffusion range of ICG on the gastric wall. Patient demographics, pathological characteristics, operative details, and data from fluorescence measurements of surgical specimens were collected. Pathological data were based on the 8th edition of the American Joint Committee on Cancer staging manual [21], and histologic data were based on the Lauren classification [22] and WHO classification [23]. This retrospective study was approved by the Institutional Review Board of Severance Hospital, Yonsei University Health System (4-2022-0826).

### 2.2. Preoperative Tumor Localization

**Figure 1:**
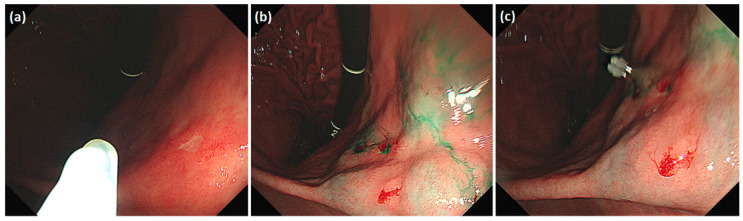
Preoperative tumor localization: (**a**) endoscopic identification of the tumor; (**b**) submucosal ICG injection around the tumor in each quadrant; (**c**) additional application of hemoclips proximal to the tumor.

Detailed endoscopic clipping procedures have been described previously [12,13], and ICG injection and hemoclip application were performed at the same time on the day before surgery. As previously reported [14,19,24,25], we endoscopically injected ICG (Dongindang Pharmaceutical Co., Siheung-si, Korea) solution into the gastric submucosal layer at four quadrants around the tumor, on the day before performing NIR fluorescence-guided surgery. Each of the four quadrants was injected with 0.6 mL of 0.625 mg/mL ICG solution for a total of 1.5 mg of ICG. In addition, endoscopic hemoclips were used to grossly delineate the tumor. Two hemoclips were placed proximal to the tumor (Figure 1).

### 2.3. Surgery

We performed laparoscopic or robotic radical gastrectomy with D1+ or D2 lymph node dissection according to the Korean Practice Guidelines for Gastric Cancer [7] and Japanese gastric cancer treatment guidelines [8]. Although all patients were diagnosed with early gastric cancer by preoperative evaluation, patients with any clinical suspicion of deeper depth of invasion or lymph node metastasis underwent D2 lymph node dissection. Surgical imaging systems equipped with an NIR fluorescence function were employed for fluorescence-guided surgery: the da Vinci Si/Xi Surgical System^®^ (Intuitive Surgical, Sunnyvale, CA, USA) and the PINPOINT^®^ endoscopic fluorescence imaging system (Novadaq, Mississauga, ON, Canada). To ensure complete dissection, we checked the fluorescence signal while performing lymph node dissection by switching between the fluorescence mode and white light mode or by using a fluorescence signal overlay image on a white light background [14].

**Figure 2:**
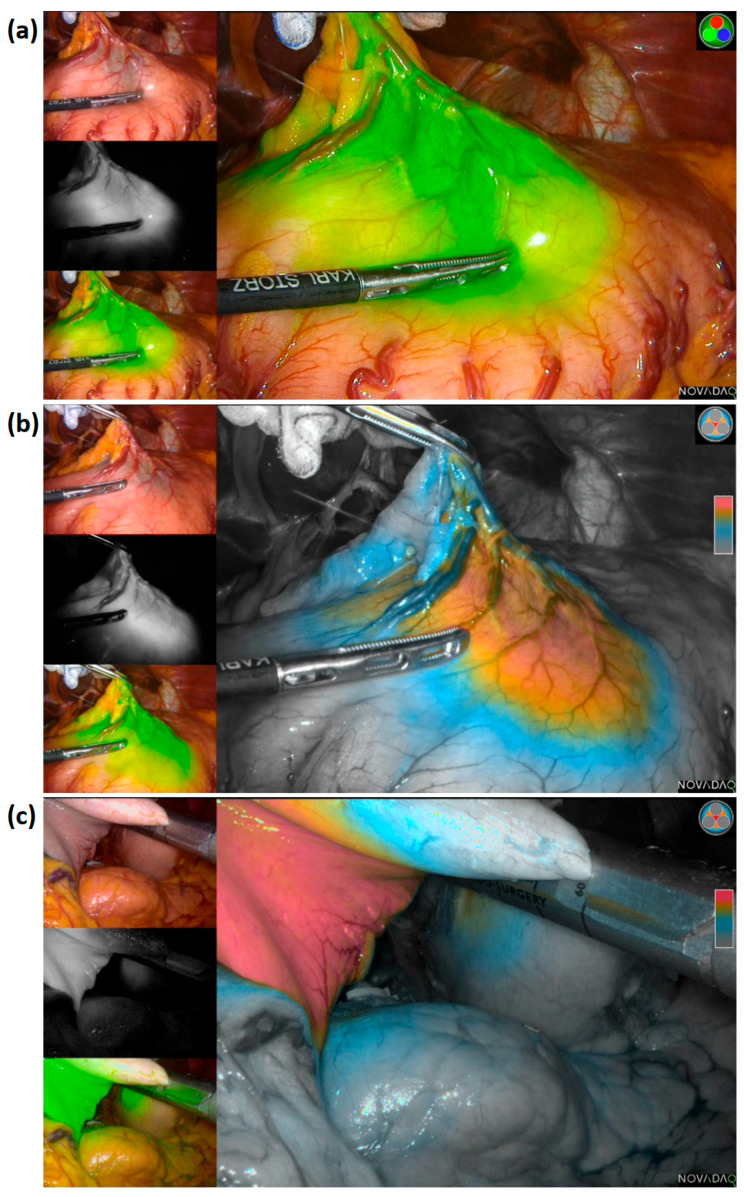
(**a**) ICG diffusion range on a gastric wall, pinpoint mode: fluorescence signal in green overlayed on the white light image-helpful to check the fluorescence image in real-time during surgery; (**b**) ICG diffusion range on a gastric wall, colorized mode: fluorescence with color gradient by signal intensity overlayed on the gray-scale white light image-helpful to check the difference in fluorescence signal intensity; (**c**) resection of the stomach with secured proximal resection margin performed by including the entire portion of ICG diffusion range in the resected specimen.

The extent of resection was determined according to the tumor location identified using the fluorescence signal from the ICG diffusion area on the gastric wall (Figure 2). As we injected ICG around the tumor before surgery, we hypothesized that the lesion was located at the center of the range of the fluorescence signal. Therefore, we decided on the gastric transection line considering the tumor location using real-time fluorescence imaging and resected the stomach proximal to the fluorescent area whenever possible. As for reconstruction methods, gastroduodenostomy, loop gastrojejunostomy, or Roux-en-Y gastrojejunostomy was considered for subtotal gastrectomy patients. Proximal gastrectomy and total gastrectomy were followed by double tract reconstruction and Roux-en-Y esophagojejunostomy. All procedures, including resection and reconstruction, were performed intracorporeally using an articulating linear stapler.

### 2.4. Specimen Examination for Margin Evaluation

**Figure 3:**
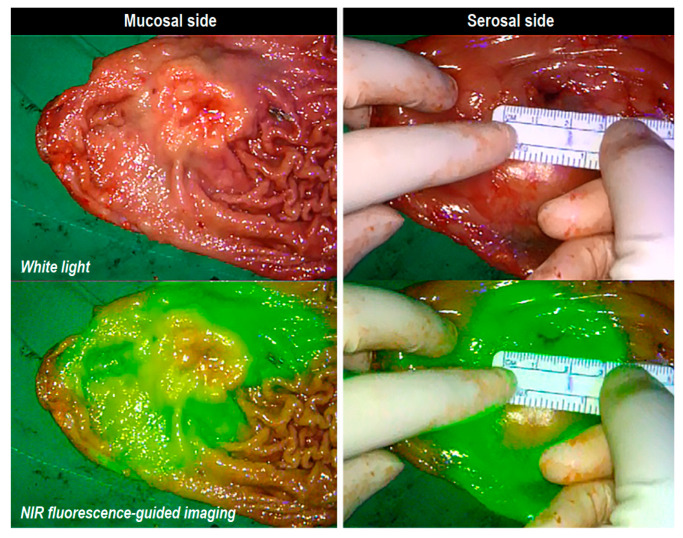
Specimen examination: measurement of the fluorescent diffusion range on a gastric specimen. (**left**) mucosal side; (**right**) serosal side.

After surgery, we immediately evaluated the surgical specimen and performed back-table dissection. Lymph node-bearing adipose tissue was separated from the gastric specimen according to the anatomical definitions of the Japanese classification of gastric carcinoma [26]. Lymph node-bearing adipose tissue was additionally evaluated using NIR fluorescence imaging for the separation of lymph nodes and basins, as previously described [14]. The gastric wall was opened along the greater or lesser curvature on the opposite side of the lesion for gross inspection. The resected stomach dimensions and extent of NIR fluorescence signal were measured (Figure 3). The long axis and orthogonal length of the ICG diffusion range were measured on both the mucosal and serosal sides of the gastric wall. Tumor size and length of the proximal or distal resection margin were measured and recorded.

### 2.5. Statistical Analysis

Statistical analyses were performed using the IBM SPSS Statistics software for Windows (version 26.0; IBM Corp., Armonk, NY, USA). Continuous variables were expressed using the mean and standard deviation or median value and interquartile range. Student’s *t*-test or the Mann–Whitney *U* test were used to compare the continuous variables. Categorical variables are presented as absolute numbers (percentages), and the chi-square or Fisher’s exact test was used. All tests were two-sided, and statistical significance was considered as a *p* value < 0.05.

## 3. Results

A total of 2235 consecutive patients were identified who underwent curative gastrectomy for EGC during the study period. Among them, we excluded 1340 patients owing to the following reasons: patients that underwent open surgery (*n* = 147), those that underwent additional surgery after endoscopic submucosal dissection (*n* = 357), those that had multiple synchronous gastric cancers (*n* = 67), those that had not received ICG injection before surgery (*n* = 616), and those that did not have recorded measurements of the diffusion range on surgical specimens after surgery (*n* = 153). In addition, we excluded 392 patients with tumors located at the cardia, fundus, incisura, and antrum/pylorus to accurately evaluate the length or status of the resection margin. Finally, 503 patients with EGC located in the gastric body were included in this study.

Of the 503 patients, 404 (80.3%) underwent subtotal gastrectomy, 68 (13.5%) underwent proximal gastrectomy, and 31 (6.2%) underwent total gastrectomy (Table 1). No preoperative ICG injection procedure- or fluorescence-guided surgery-related complications were reported. The mean tumor size was 26.9 ± 17.0 mm (Table 2). The lengths of the proximal and distal resection margins were 35.0 ± 20.6 mm (range, 0–130 mm) and 88.1 ± 38.4 mm (range, 1–223 mm), respectively. There was proximal margin involvement in three patients but no distal margin involvement. In all three patients with proximal margin involvement, both the ICG fluorescent diffusion range and the hemoclips were resected, and the lengths of the proximal margin evaluated grossly by the surgeon were 10, 40, and 30 mm, respectively, and those evaluated by the pathologist were 8, 25, and 5 mm, respectively (Table 3). This is because the tumor size was underestimated at preoperative diagnosis owing to the limitations of the diagnostic modality; the tumor sizes estimated preoperatively by the endoscopist were 13, 15, and 18 mm, respectively. However, on pathological examination, they were determined to be 63, 95, and 65 mm, respectively. On analysis, a mean discrepancy of 9.9 ± 10.8 mm was found between the size of lesions in endoscopic/surgical specimens and size of lesions on pathological examination results, and 16 out of 503 patients had extreme outliers (size discrepancy >3rd quartile + 3 x interquartile range; >39 mm). Three cases of proximal margins being involved by the tumor were included among the extreme outliers, showing size discrepancies of 75, 56, and 45 mm. The size of one of the remaining 13 cases was overestimated in the preoperative examination compared to its actual size on pathological examination. In five cases with unclear tumor margins, ICG injection and gastric resection were conservatively performed. In one of these five cases, resection margin evaluation was performed with a frozen section during surgery. In other cases, the tumor size was underestimated; however, negative resection margins could be achieved by securing an average distance of 29.2 mm (range: 1–95 mm) from the proximal margin.

The ICG diffusion range through the gastric wall had a long axis of 82.7 ± 23.8 mm and a short axis of 75.3 ± 21.2 mm on the mucosal side, and a long axis of 86.7 ± 23.2 mm and a short axis of 80.2 ± 21.6 mm on the serosal side. The larger the tumor, the greater the range of ICG diffusion on both the mucosal and serosal sides of the gastric wall (Table 4).

The length of the resection margin was analyzed according to the extent of resection and subsequent reconstruction type (Table 5). In the patient who underwent distal gastrectomy, the length of the proximal resection margin was 35.9 ± 20.3 mm. The proximal resection margin lengths for gastroduodenostomy, loop gastrojejunostomy, and Roux-en-Y gastrojejunostomy were 38.1 ± 20.1, 33.4 ± 22.2, and 28.7 ± 17.2 mm, respectively. The distal resection margin lengths for gastroduodenostomy, loop gastrojejunostomy, and Roux-en-Y gastrojejunostomy were 38.1 ± 20.1, 33.4 ± 22.2, and 28.7 ± 17.2 mm, respectively. The length of the proximal resection margin in proximal and total gastrectomy patients was 50.8 ± 33.8 and 124.8 ± 33.9 mm, respectively. The length of the distal resection margin in proximal and total gastrectomy patients was 50.8 ± 33.8 and 124.8 ± 33.9 mm, respectively.

## 4. Discussion

In this study, submucosal ICG injection for fluorescence-guided lymphadenectomy during gastrectomy could secure a resection margin of 28 mm or greater when the entire diffusion range of ICG under NIR imaging was resected. Determining the resection margin based on the ICG diffusion range is a simple and time-saving technique because it is perceptive under NIR imaging and does not require additional intraoperative maneuvers.

For curative resection, it is recommended to secure a resection margin of ≥3 cm for expansive growth pattern, ≥5 cm for infiltrative types of T2 tumors or deeper, and ≥2 cm for T1 cancer. The extent of gastrectomy and reconstruction was determined according to the location of the proximal and distal resection lines. The extent of resection and the reconstruction method have a crucial effect on morbidity and mortality in the early postoperative phase and on nutritional outcomes and quality of life in the later postoperative stage [27,28,29]. Exact tumor localization has important implications for gastrectomy because it may increase the chances of organ preservation; that is, a partial gastrectomy can be performed instead of a total gastrectomy if the tumor location is properly identified [30].

The lack of change in the serosal surface of early gastric cancer makes it challenging to decide the resection line during surgery according to the tumor location. During minimally invasive surgery, it is more difficult to locate the tumor owing to a lack of tactile sensation. Various tumor localization methods have been devised in this context; the simplest method is intraoperative endoscopy, which can directly visualize lesions. It has the advantages of eliminating a separate preoperative endoscopic localization procedure and offering the additional use of endoscopy to confirm the completeness of the anastomosis. However, intraoperative endoscopic procedures increase operation time. Additionally, the operating room must contain endoscopic equipment, and a separate endoscopist is required during surgery. Moreover, exact estimations of the resection margin during intraoperative endoscopic examination are not always feasible, because applying a stapler may obstruct endoscopic visualization of the lesion.

To identify the location of the lesion more simply without an intraoperative endoscopic procedure, endoscopic hemoclips were applied along with preoperative endoscopy. The clips were then identified by intraoperative simple radiography or by ultrasound [12,13]. Although this method requires an endoscopic procedure before surgery, if clips are applied to a patient who is expected to undergo surgery during diagnostic gastroduodenoscopy, the surgery can be continued without interruption with an additional endoscopic procedure. However, the additional intraoperative process of taking a portable X-ray to detect the endoscopic clips prolongs the operation time, although it is expected to be shorter than that of an intraoperative endoscopic procedure. More importantly, the accurate location of the clips cannot be identified in real time when resecting the stomach, because they cannot be visualized on the serosal surface.

Endoscopic tattooing by injecting a dye or autologous blood is another method that ensures that the location is always marked on the serosal surface of the gastrointestinal tract without any additional manipulation during surgery [31,32]. This technique also requires an endoscopic procedure before surgery; however, additional equipment or procedures are not required during the surgery. An advantage is that the injection site is always visible; however, it is difficult to identify the exact location of the lesion because of the invariable range of the marked area. In addition, the surgical field is obscured by the opaque dye or blood stains in the area around the injection site. Thus, endoscopic tattooing presents a problem, as it is difficult to standardize the procedure, such as how to inject, when to inject, what dose to inject, and how to determine the extent of the spread of dye.

ICG has also been used as an alternative to other tattooing dyes [33,34]. Although an endoscopic procedure is required before surgery, ICG injection does not require additional time and equipment during surgery, unlike other tumor localization methods. Recently, ICG injection has been used for fluorescence lymphography, not for tumor localization, to visualize draining lymphatics and lymph nodes under NIR imaging during minimally invasive gastrectomy. NIR fluorescence lymphography-guided gastrectomy has shown beneficial effects in identifying and retrieving draining lymph nodes from the tumor and in the consequential complete dissection of the lymph nodes [14,19,24,25]. In addition, without any procedures, NIR fluorescence-guided gastrectomy has the potential to provide tumor localization and secure a sufficient resection margin. Since the ICG diffusion area during fluorescence lymphography-guided gastrectomy is not detectable in the visible spectrum and only visible on NIR imaging, ICG diffusion does not obscure the surgical field and does not interfere with surgery. Selective and repeated images or an image overlay of NIR fluorescence help visualize the ICG diffusion area in real time during surgery, and this overcomes the disadvantage of one-time tumor localization of other localization methods. Furthermore, ICG injection for NIR fluorescence-guided surgery could provide sufficient information to secure the resection margin in gastric cancer surgery in patients with EGC.

There are limitations to the ICG injection technique in terms of tumor localization and resection margin determination. Unlike endoscopic clip application, it is challenging to identify the exact location of the injection site or the tumor, and failure owing to technical problems, such as ICG spillage or insufficient diffusion, is possible. Therefore, applying endoscopic clips at the proximal margin of the tumor, as performed in our technique, or checking the intraluminal location of the tumor with intraoperative gastroduodenoscopy could be a supplementary measure for tumor localization during NIR fluorescence-guided surgery. As the injected ICG is washed out over time and does not remain in the stomach wall for more than two days, it cannot be applied if an endoscopy cannot be performed on the day before surgery. The duration or degree of diffusion remaining on the stomach wall varies depending on the ICG dose or concentration. Research on the diffusion range according to the dose and concentration of ICG is required to establish a standardized injection protocol. However, because the dose and concentration of ICG are optimized for lymphography, adjustment of the dose and concentration of ICG is not feasible and cannot be performed for tumor localization. Ultimately, it is necessary to develop a method that can accurately identify the location of the tumor in real time from the serosal side without obscuring the surgical field, regardless of the timing of tumor localization.

This study has some limitations. As it is a retrospective study, data on the timing of endoscopic ICG injection, surgery, and ICG diffusion range measurement were unavailable. Therefore, the difference in the ICG diffusion range according to the time duration after ICG injection could not be evaluated in this study. Additionally, data on the detailed location of the tumor in the ICG diffusion range were unavailable as well. Further studies with analysis of differences in the ICG diffusion range over time and detailed information on the diffusion of injected ICG according to tumor characteristics, location, and orientation in the gastric wall will help expand the evidence database on the efficacy of the ICG diffusion range in determining resection extent during gastrectomy.

## 5. Conclusions

The ICG diffusion range during NIR fluorescence lymphography-guided gastrectomy provides a collateral effect of simple, safe, and constant tumor localization and consequently secures an adequate resection margin for patients with EGC. ICG injection for NIR fluorescence lymphography-guided gastrectomy is not only an effective tool for visualizing draining lymphatics and lymph nodes, but it is also a useful technique for determining the resection margin.

## Figures and Tables

**Table 1:** Patient characteristics.

Variable	Total (n = 503)
Age (years)	57.0 ± 11.3
Sex (*n*, %)	
Male	220 (43.7%)
Female	283 (56.3%)
BMI	23.3 ± 2.9
ASA-PS classification (*n*, %)	
ASA I	78 (15.5%)
ASA II	315 (62.6%)
ASA III	107 (21.3%)
ASA IV	3 (0.6%)
Tumor location, Tubular (*n*, %)	
Lower body	262 (52.1%)
Midbody	153 (30.4%)
Upper body	86 (17.1%)
Lower body~Midbody	1 (0.2%)
Midbody~Upper body	1 (0.2%)
Tumor location, Circular (*n*, %)	
Lesser curvature	141 (28.0%)
Greater curvature	102 (20.3%)
Anterior wall	101 (20.1%)
Posterior wall	159 (31.6%)
Operation method (*n*, %)	
Laparoscopic	235 (46.7%)
Robotic	268 (53.3%)
Gastrectomy type (*n*, %)	
Subtotal gastrectomy	404 (80.3%)
Proximal gastrectomy	68 (13.5%)
Total gastrectomy	31 (6.2%)
Reconstruction (*n*, %)	
Gastroduodenostomy	275 (54.7%)
Gastrojejunostomy, loop	66 (13.1%)
Roux-en-Y gastrojejunostomy	63 (12.5%)
Double tract reconstruction	68 (13.5%)
Roux-en-Y esophagojejunostomy	31 (6.2%)
LN dissection extent (*n*, %)	
D1+	483 (96.0%)
D2	20 (4.0%)
Blood loss (mL)	63.0 ± 197.0
Operation time (min)	177.1 ± 51.5
Length of hospital stay (days)	5.7 ± 3.9

NOTE. Data are presented as mean ± standard deviation or as a number (percentage). Abbreviations: ASA-PS, The American Society of Anesthesiologists (ASA) physical status; BMI, body mass index.

**Table 2:** Pathological results.

Variable	Total (n = 503)
Histology, Lauren classification (*n*, %)	
Intestinal	338 (67.2%)
Diffuse	112 (22.3%)
Mixed	34 (6.8%)
Other	19 (3.8%)
Histology, WHO classification (*n*, %)	
Tubular denocarcinoma	
- Well differentiated	26 (5.2%)
- Moderately differentiated	101 (20.1%)
- Poorly differentiated	121 (24.1%)
Poorly cohesive carcinoma	
- Signet-ring cell carcinoma	111 (22.1%)
- Other	133 (26.4%)
Mucinous adenocarcinoma	2 (0.4%)
Mixed adenocarcinoma	2 (0.4%)
Carcinoma with lymphoid stroma	4 (0.8%)
Others	3 (0.6%)
Depth of invasion (*n*, %)	
T1a (mucosa)	291 (57.9%)
T1b (submucosa)	164 (32.6%)
T2 (muscularis propria)	29 (5.8%)
T3 (subserosa)	17 (3.4%)
T4a (serosa)	1 (0.2%)
Tx (cannot be assessed)	1 (0.2%)
Regional nodal involvement (*n*, %)	
N0 (none)	458 (91.1%)
N1 (1 or 2 lymph nodes)	20 (4.0%)
N2 (3 to 6 lymph nodes)	15 (3.0%)
N3a (7 to 15 lymph nodes)	7 (1.4%)
N3b (16 or more lymph nodes)	2 (0.4%)
Nx (cannot be assessed) *	1 (0.2%) *
No. of metastatic LN (*n*, %)	0.5 ± 4.0
No. of total LN (*n*, %)	44.6 ± 16.3
Tumor size (mm)	26.9 ± 17.0
Length of proximal margin (mm)	35.0 ± 20.6
Involvement of proximal margin (*n*, %)	3 (0.6%)
Length of distal margin (mm)	88.1 ± 38.4
Involvement of distal margin (*n*, %)	0 (0%)

* The nodal status could not be assessed in one patient because of perigastric lymph node involvement due to concurrent follicular lymphoma.

**Table 3:** Characteristics of three patients with extension of carcinoma into proximal resection margin.

Variable	Case #1	Case #2	Case #3
Sex/Age	F/66	M/54	M/48
Tumor location	LB/LC	LB/GC	LB/LC
Endoscopic finding	EGC, type IIc, 13 mm	EGC, type IIc, 30 mm	EGC, type IIa + IIc, 18 mm
Endoscopic image	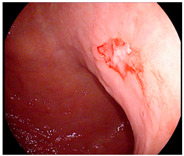	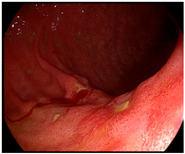	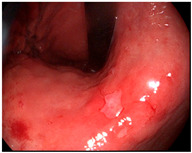
EUS	Submucosa+, 13 mm, LN (-)	N/A	Submucosa+, 21 mm, LN (-)
Helicobacter pylori	Positive	N/A	Positive
Preoperative histology	Signet ring cell carcinoma	Adenoca., moderate diff.	Adenoca., moderate diff.
Surgery	Robot STG BI	Robot-attempted STG BII	Robot STG BI
Specimen gross evaluation by the surgeon	Lesion 20 × 20 mm	Lesion 15 × 10 mm	Lesion 20 × 20 mm
	Proximal margin 10 mm	Proximal margin 40 mm	Proximal margin 30 mm
	Distal margin enough	Distal margin 80 mm	Distal margin 60 mm
	ICG (mucosa) 70 × 60 mm	ICG (mucosa) 90 × 80 mm	ICG (mucosa) 60 × 60 mm
	ICG (serosa) 80 × 80 mm	ICG (serosa) 80 × 70 mm	ICG (serosa) 70 × 70 mm
Specimen gross evaluation by the pathologist	Lesion 30 × 15 mm	Lesion 15 × 10 mm	Lesion 65 × 55 mm
	Proximal margin 8 mm	Proximal margin 25 mm	Proximal margin 5 mm
	Distal margin 92 mm	Distal margin 90 mm	Distal margin 30 mm
Pathological result	Diffuse type (PCC:SRC) Invades mucosa (pT1a) Tumor size 63 × 43 mm Proximal margin involved Distal margin 66 mm Lymph node 0/33 (pN0)	Intestinal type (AMD with crawling features) Invades submucosa, sm1 (pT1b) Tumor size 90 × 50 mm Proximal margin involved Distal margin 90 mm Lymph node 0/28 (pN0)	Intestinal type (AMD) Invades submucosa, sm1 (pT1b) Tumor size 65 × 55 mm Proximal margin involved Distal margin 30 mm Lymph node 0/38 (pN0)

Abbreviations: AMD, adenocarcinoma, moderately differentiated; BI, Billroth-I anastomosis (gastroduodenostomy); BII, Billroth-II anastomosis (loop gastrojejunostomy); EGC, early gastric cancer; GC, greater curvature; ICG, indocyanine green; LB, lower body; LC, lesser curvature; LN, lymph node; PCC, poorly cohesive carcinoma; SRC, signet ring cell carcinoma; STG, subtotal gastrectomy.

**Table 4:** Tumor size and ICG diffusion range on gastric wall.

Variable	Total (n = 503)	Tumor < 2 cm (n = 187)	Tumor, 2–4 cm (n = 225)	Tumor ≥ 4 cm (n = 91)
Tumor size (mm)	26.9 ± 17.0 × 19.0 ± 12.8	12.4 ± 4.2 × 9.0 ± 3.7	27.1 ± 5.5 × 19.0 ± 6.0	55.3 ± 15.5 × 39.1 ± 13.0
ICG diffusion range, mucosal side (mm)	82.7 ± 23.8 × 75.3 ± 21.2	81.1 ± 23.9 × 73.9 ± 21.2	82.7 ± 24.2 × 76.0 ± 20.3	85.9 ± 22.4 × 76.5 ± 23.5
ICG diffusion range, serosal side (mm)	86.7 ± 23.2 × 80.2 ± 21.6	85.7 ± 23.8 × 77.9 ± 21.1	86.2 ± 22.3 × 81.8 ± 21.1	89.6 ± 23.9 × 80.9 ± 23.5

**Table 5:** Length of proximal or distal resection margin on a pathology specimen.

Variable	n (%)	Proximal Margin (mm)	Distal Margin (mm)	Tumor Size (mm)	ICG Diffusion Range, Mucosal Side (mm)	ICG Diffusion Range, Serosal Side (mm)
Total gastrectomy	31 (6.2%)	34.9 ± 24.5	124.8 ± 33.9	41.2 ± 24.2 × 29.8 ± 18.4	98.5 ± 36.5 × 82.6 ± 32.3	101.8 ± 30.6 × 87.1 ± 33.1
Proximal gastrectomy	68 (13.5%)	29.7 ± 19.6	50.8 ± 33.8	27.3 ± 18.5 × 20.3 ± 13.4	78.8 ± 26.8 × 72.6 ± 20.5	85.4 ± 27.3 × 87.1 ± 33.1
Subtotal gastrectomy	404 (80.3%)	35.9 ± 20.3	91.4 ± 34.9	25.7 ± 15.5 × 18.0 ± 11.7	82.1 ± 21.5 × 75.2 ± 20.2	85.7 ± 21.4 × 79.8 ± 20.2
Gastroduodenostomy	275	38.1 ± 20.1	79.5 ± 27.6	24.4 ± 14.1 × 17.0 ± 10.9	79.8 ± 21.2 × 74.8 ± 19.4	83.0 ± 20.4 × 78.9 ± 19.1
Loop gastrojejunostomy	66	33.4 ± 22.2	108.5 ± 36.9	26.8 ± 19.0 × 18.8 ± 12.1	84.8 ± 23.1 × 71.9 ± 21.1	88.3 ± 20.7 × 80.2 ± 23.2
Roux-en-Y gastrojejunostomy	63	28.7 ± 17.2	125.5 ± 31.7	30.0 ± 16.9 × 21.4 ± 14.2	89.4 ± 19.5 × 80.5 ± 21.9	94.9 ± 23.6 × 83.6 ± 21.0

## Data Availability

The data presented in this study are available upon request from the corresponding author.
